# RhoA Activation Sensitizes Cells to Proteotoxic Stimuli by Abrogating the HSF1-Dependent Heat Shock Response

**DOI:** 10.1371/journal.pone.0133553

**Published:** 2015-07-20

**Authors:** Roelien A. M. Meijering, Marit Wiersma, Denise M. S. van Marion, Deli Zhang, Femke Hoogstra-Berends, Anne-Jan Dijkhuis, Martina Schmidt, Thomas Wieland, Harm H. Kampinga, Robert H. Henning, Bianca J. J. M. Brundel

**Affiliations:** 1 Department of Clinical Pharmacy and Pharmacology, University of Groningen, University Medical Center Groningen, Groningen, The Netherlands; 2 Department of Cell Biology, University of Groningen, University Medical Center Groningen, Groningen, The Netherlands; 3 Department of Molecular Pharmacology, University of Groningen, Groningen, The Netherlands; 4 Institute of Experimental and Clinical Pharmacology and Toxicology, Mannheim Medical Faculty, University of Heidelberg, Mannheim, Germany; 5 Department of Physiology, Institute for Cardiovascular Research, VU University Medical Center, Amsterdam, The Netherlands; Boston University Medical School, UNITED STATES

## Abstract

**Background:**

The heat shock response (HSR) is an ancient and highly conserved program of stress-induced gene expression, aimed at reestablishing protein homeostasis to preserve cellular fitness. Cells that fail to activate or maintain this protective response are hypersensitive to proteotoxic stress. The HSR is mediated by the heat shock transcription factor 1 (HSF1), which binds to conserved heat shock elements (HSE) in the promoter region of heat shock genes, resulting in the expression of heat shock proteins (HSP). Recently, we observed that hyperactivation of RhoA conditions cardiomyocytes for the cardiac arrhythmia atrial fibrillation. Also, the HSR is annihilated in atrial fibrillation, and induction of HSR mitigates sensitization of cells to this disease. Therefore, we hypothesized active RhoA to suppress the HSR resulting in sensitization of cells for proteotoxic stimuli.

**Methods and Results:**

Stimulation of RhoA activity significantly suppressed the proteotoxic stress-induced HSR in HL-1 atrial cardiomyocytes as determined with a luciferase reporter construct driven by the HSF1 regulated human HSP70 (HSPA1A) promoter and HSP protein expression by Western Blot analysis. Inversely, RhoA inhibition boosted the proteotoxic stress-induced HSR. While active RhoA did not preclude HSF1 nuclear accumulation, phosphorylation, acetylation, or sumoylation, it did impair binding of HSF1 to the *hsp* genes promoter element HSE. Impaired binding results in suppression of HSP expression and sensitized cells to proteotoxic stress.

**Conclusion:**

These results reveal that active RhoA negatively regulates the HSR via attenuation of the HSF1-HSE binding and thus may play a role in sensitizing cells to proteotoxic stimuli.

## Introduction

The heat shock response (HSR) is one of the main pro-survival stress responses of the cell, restoring cellular homeostasis upon exposure to proteotoxic stimuli, including heat shock, oxidative stress, heavy metal exposure, and inhibition of the proteasome [1–3]. The primary targets of the HSR are heat shock genes that encode heat shock proteins (HSPs), which act as molecular chaperones that assist in the refolding and degradation of damaged proteins [3,4]. Heat shock transcription factor 1 (HSF1) activity is the main factor governing the HSR [2,5]. HSF1 activation is a multistep process that is negatively regulated by chaperones, including HSPCA (HSP90), HSPA1A (HSP70) [1], and TRiC [6]. Upon heat shock, monomeric HSF1 converts to a trimer that accumulates in the nucleus and subsequently binds to the heat shock element (HSE) within the promoter region of *hsp* genes [2]. In addition, extensive posttranslational modifications such as phosphorylation, acetylation, and sumoylation are thought to fine-tune HSF1 activity [2,5,7]. Failure to mount an adequate HSR is thought to underlie hypersensitivity to acute proteotoxic stress and has been associated with disease progression in age-related chronic protein aggregation diseases, such as Huntington’s, Alzheimer’s, and Parkinson’s disease, and shortening of life-span [2,3]. Atrial fibrillation represents another age-related progressive disease in which cardiac cells fail to mount an adequate HSR in response to stress caused by rapid electrical stimulation [8]. Hereby the accumulation of protein damage that impedes cell function and survival is stimulated [8–10]. Importantly, priming the HSR in cardiac cells by geranylgeranylacetone pretreatment or the single overexpression of the HSF1 target gene *hspb1* was found to maintain proper function in rapidly paced cells [8,11,12]. Why cardiac cells are unable to mount a proper HSR in response to atrial fibrillation is unknown. Activation of the Ras homolog gene family member A (RhoA) serves a possible candidate. RhoA represents a major stress signaling pathway, which was previously found to become activated during the progression of atrial fibrillation [12–14]. Moreover, we observed that the cardioprotective effects of small HSPB family members in atrial fibrillation were accompanied by the attenuation of the RhoA signaling [12]. The activation of RhoA is controlled by three classes of regulatory proteins, i.e. GTPase-activating proteins (GAPs), guanine nucleotide dissociation inhibitors (GDIs), and guanine nucleotide exchange factors (GEFs). GAPs and GDIs inactivate RhoA by promoting the GDP-bound state and GEFs activate RhoA by stimulating the exchange of GDP for GTP. RhoA signaling, primarily through its downstream effector RhoA kinase (ROCK), regulates a wide variety of cellular functions, including cytoskeleton reorganization, cell cycle progression, gene expression, and cell death [15,16]. We hypothesized that RhoA signaling may negatively regulate the HSR. Consistent with this hypothesis, we show that active RhoA is a suppressor of the HSR by impairing the HSF1 binding to the HSE, consequently resulting in the inhibition of HSP expression and hyper-sensitization of cells to proteotoxic stress.

## Materials and Methods

### Cell culture

HL-1 adult mouse-derived atrial cardiomyocytes were obtained from Dr. William Claycomb [17] as described before [8]. The cardiomyocytes were maintained in complete Claycomb medium (JRH, UK) supplemented with 100 μM norepinephrine (Sigma, The Netherlands), 0.3 mM L-ascorbic acid (Sigma, The Netherlands), 2 mM L-glutamine (Gibco, The Netherlands), 100U/ml penicillin (PAA Laboratories GmbH), 100 μg/ml streptomycin (PAA Laboratories GmbH), and 10% FBS (Sigma, The Netherlands). They were cultured on 12.5 mg/ml fibronectin (Sigma, The Netherlands) and 0.02% gelatin (Sigma, The Netherlands) coated surfaces.

Human embryonic kidney 293 (HEK-293) cells were grown in Dulbecco modified Eagle medium (Gibco) supplemented with, 100U/ml penicillin (PAA Laboratories GmbH), 100 μg/ml streptomycin (PAA Laboratories GmbH), and 10% FBS (Sigma, The Netherlands). Cells were grown at 37°C in 5% CO_2_.

Cell viability was measured 4h after heat shock by staining the cells with trypan blue (1:1), followed by counting of the unstained (viable) and stained (dead) cells in a Burker Turk counting chamber.

### Constructs

Constructs used in this study were: pcDNA3.1+ (empty plasmid, Invitrogen), and the reporter plasmids, pSRE-luc, to monitor RhoA activity [18], or pGL3-HSPA1A-luciferase, to monitor HSPA1A expression [19]. pCDNA5-FRT-TO-RhoA-WT (wild type) and pCDNA5-FRT-TO-C3T (C3-transferase, C3-exoenzyme) were constructed by PCR of the RhoA-WT and C3-exoenzyme coding sequences from pRK5-RhoA and pEF-myc-C3T [20]. The RhoA-v14 and RhoA-n19 mutant constructs were obtained from Addgene (USA). PDM2-LacZ plasmid was kind gift from Dr. B. Eggen (UMCG, The Netherlands). Primers used are: C3T fw; GAGGACCTGGGATCCTCTAG, C3T rv; CGGCTCGCCGGCCGCTCATTGCCATATATTGGGTATAAATAGC, RhoA fw; GACCTGGGATCCATGGCTGCCATCCGGAAGAAAC, RhoA rev; AAATATCGCGGCCGCTCACAAGACAAGGCACCCAG. Coding sequences and pCDNA5-FRT-TO plasmids were digested with BamHI and NotI and subsequently ligated to obtain pCDNA5-FRT-TO-RhoWT and pCDNA5-FRT-TO-C3T, respectively. The pS230A, pS303/p307A and pS303/S307D HSF1 mutants were a kind gift from of Prof. Dr. L Sistonen [21].

### Antibodies, chemical compounds and transfection reagent

Antibodies used in this study were: HSPA1A (Stressgen, USA), HSF1 (Cell Signaling Technology, USA), eIF2α (Abcam, UK), eIF2α-S51P (Cell Signaling Technology, USA), Acetylated-lysine (Cell Signaling Technology, USA), SUMO1 (R&D systems, The Netherlands), SUMO2-3 (Millipore, The Netherlands), cleaved caspase-3 (Cell Signaling), RhoA (Santa-Cruz Biotechnology, The Netherlands), and GAPDH (Fitzgerald industries international, USA). Horseradish peroxidase-conjugated anti-mouse, anti-rabbit (Santa-Cruz Biotechnology, The Netherlands), and anti-goat (Dako Cytomation, Denmark) were used as secondary antibodies. Reagents used in this study are: calpeptin (Cytoskeleton, USA), MG-132 (M7449, Sigma Aldrich, The Netherlands), PD15606 (Calbiochem, The Netherlands) and HSF1 activator geranylgeranylacetone (GGA, 10 μM, Eisai, Japan). Transient (co)-transfections were performed by the use of Lipofectamin 2000 (Life technologies, The Netherlands).

### Heat shock and compound treatment

Cells were heat shocked at 45°C for 10 minutes. RhoA activity was modulated by treatment of cardiomyocytes with calpeptin, according to manufacturer’s instructions. Briefly, calpeptin was dissolved in DMSO. Cells were serum deprived on 1% FBS supplemented Claycomb medium for 16h and subsequent serum deprived on 0% FBS supplemented Claycomb medium for 24h. Cells were treated with calpeptin, 1 U/ml for 20 min, to induce RhoA activity. ROCK inhibition was achieved by Y27632 (Sigma, The Netherlands) treatment, 10 μM for 16h, or H1152P (Calbiochem), 10 nM and 100 nM for 24h. Proteasome inhibition was achieved by MG-132 (50 μM) pretreatment for 20 min. Calpain inhibition was achieved by PD15606 (20 μM) pre-treatment for 1h. In case of heat shock treatment, heat shock was applied during the last 10 minutes of compound treatment. After heat shock, cells received fresh serum free medium and were harvested at the below indicated recovery periods. Cardiomyocytes that were used for HSF1 acetylation, sumoylation, translocation, and DNA binding experiments were harvested after a 10 min recovery period after heat shock, whereas cells used for cleaved caspase-3 levels were harvested after a 2h recovery period, and cells used for HSP expression (protein and mRNA) were harvested after a 4h recovery period.

### Luciferase assay

Luciferase assays were performed 48 hours after transfection and 4h after heat shock. HL-1 cardiomyocytes were lysed and scraped in BLUC (25 mM Tris/H_3_PO_4_ (pH 7.8), 10 mM MgCl_2_, 1% (v/v) Triton X-100, 15% glycerol, and 1 mM EDTA). Luciferase activity in the samples was measured for 10 s after injecting the substrate buffer (BLUC, 1.25 mM ATP, and 0.087 mg/ml D-luciferin) in a Wallac 1420 Victor3 V plate reader. Transient transfection efficiency was determined by co-transfection of cells with pC, pC3T, pRhoA-WT or HSF1 mutant construct with a β-galactosidase construct (PDM2-LacZ, kind gift from Dr. B. Eggen, UMCG, The Netherlands). To measure the β-galactosidase activity, cells were lysed and scraped in BLUC (25 mM Tris/H3PO4 (pH 7.8), 10 mM MgCl2, 1% (v/v) Triton X-100, 15% glycerol and 1 mM EDTA). Luciferase activity in the samples was measured for 1s after injecting the substrate buffer (BLUC, 1.25 mM ATP and 0.087 mg/ml D-luciferin). β-Galactosidase activity was determined in 100 mM Na2HPO4/NaH2PO4, 1 mM MgCl2, 100 mM 2-mercaptoethanol, and 0.67 mg/ml O-nitrophenylgalactopyranoside, incubated 4h-overnight and measured at 405 nm (Wallac 1420 plate reader).

### G-LISA RhoA activity measurement

For the quantitative analysis of active RhoA GTP levels, GLISA RhoA Activation Assay (Cytoskeleton, USA) was performed according to the manufacturer’s instructions. Briefly, after drug treatment, cardiomyocytes were harvested in Rho-GLISA lysis buffer (supplied). After measurement of the protein concentration with the use of Precision Red (supplied), equal amounts of protein were incubated in RhoA-GTP affinity plates. The amount of bound RhoA-GTP was detected by using primary anti-RhoA antibody (supplied) and secondary HRP-labeled antibody (supplied). Subsequently, samples were incubated with HRP detection reagent for 15 min after which a HRP stop buffer was added (supplied). Colorimetric detection at 490 nm was performed immediately in a BioRad Benchmark plus microplate-reader (BioRad, The Netherlands).

### Isolation of cytosolic and nuclear fractions

Cytosolic and nuclear fractions were obtained by harvesting the cardiomyocytes in membrane lysis buffer (10 mM Hepes (pH 8.0), 1.5 mM MgCl_2_, 10 mM KCl, 1 mM DTT, and 1% v/v Igepal-CA630). After centrifugation, the supernatant (cytosolic fraction) was transferred to an eppendorf tube and the pellet was resuspended in nuclear envelope lysis buffer (20 mM Hepes (pH 8.0), 1.5 mM MgCl_2_, 25% v/v glycerol, 0.42 M NaCl, 0.2 mM EDTA, and 1 mM DTT) to obtain the nuclear fraction.

### Protein-extraction and Western Blot analysis

Standard protein-extraction was performed with RIPA lysis buffer. Western Blot analysis was performed as described previously [11]. Briefly, equal amounts of protein in SDS-PAGE sample buffer were homogenized by use of a 26G needle and syringe, before separation on 4–20% PAA-SDS gels (Thermo Scientific, USA). After transfer to nitrocellulose membranes (Stratagene, The Netherlands), membranes were incubated with primary antibodies and subsequently Horseradish peroxidase-conjugated anti-mouse, anti-rabbit or anti-goat was used as secondary antibody depending on the origin of the primary antibody. Signals were detected by the SuperSignal-detection method (Thermo Scientific, USA) and quantified by densitometry (GeneGnome/GeneTools from SynGene, USA).

### Immunoprecipitation

Nuclear fractions were preincubated with A/G agarose beads (Santa Cruz Biotechnology, USA) for 1 hour at 4°C to remove proteins that nonspecifically attach to the beads. After centrifugation, input fractions were made by adding 6x SDS sample buffer to 50 μg protein. IP fractions were prepared by incubating equal amounts of protein with 5 μl HSF1 primary antibody for 2 hours at 4°C, after which 30 μl of A/G agarose beads were added and incubated for 3 hours at 4°C. The protein-bead complexes were washed 4 times with immunoprecipitation buffer and bound proteins were removed from the beads by boiling for 5 minutes in 2x buffer. Then, the input samples were applied for Western blot analysis as described above.

### Immunofluorescent staining and confocal analysis

Cardiomyocytes were grown on fibronectin 12.5 mg/ml fibronectin (Sigma) and 0.02% gelatine (Sigma) coated glass coverslips. After drug treatment as described above, cardiomyocytes were fixated with 4% paraformaldehyde for 15 minutes, washed three times with Phosphate-Buffered Saline (PBS), and blocked and permeabilized for 60 min in 5% BSA, 0.3% Triton X-100 in PBS. Samples were subsequently incubated overnight with HSF1 antibody 1:100 (Cell Signaling Technology) in 1% BSA, 0.3% Triton X-100 in PBS. Fluorescein labeled isothiocyanate (FITC) anti-rabbit (Jackson ImmunoResearch, The Netherlands) was used as secondary antibody 1:200 in combination with TOTO-3 iodide, 2 μM (Life technologies, The Netherlands) as a nuclear counterstain. Cells were mounted in Vectashield without DAPI (Vector Laboratories, USA) and analyzed using an AOBS Leica confocal microscope.

### Quantitative Real Time-PCR analysis

Total RNA from HL-1 cardiomyocytes was extracted using the RNA extraction kit Nucleospin II (Machery-Nagel, Germany). cDNA synthesis was performed according to standard methods. Briefly, first strand cDNA was synthesized using random primer mix (Promega, USA) and subsequently used (1 μg per reaction) as a template for quantitative real-time reverse-transcriptase PCR (qRT-PCR). All mRNA levels were expressed in relative units on the basis of a standard curve (serial dilutions of a calibrator cDNA mixture). All PCR results were normalized against GAPDH. All reactions were made in triplicates with samples derived from three biological repeats. The sequences for the primers were as follows: fw; CATCAAGAAGGTGGTGAAGC, rv; ACCACCCTGTTGCTGTAG for HSPA1A, fw; ATCTTTGGTTGCTTGTCGCT, rv; ATGAAGGAGACTGCTGAGGC for HSPA5, fw; TGTATTTCCGGGTGAAGCAC, rv; CAGTGAAGACCAAGGAAGGC for HSPB1, fw; TGACTTTGCAACAGTGACCC, rv; GCTGTAGCTGTTACAATGGGG for HSPD1, fw; TCCGTGGAATGTGTAGCTGA, rv; GATTTTCGACCGCTATGGAG for DNAJB1, fw; ATTGGTTGGTCTTGGGTCTG, rv; GCCAGTTGCTTCAGTGTCCT for HSPCA, and fw; GCAAGGAGAAGCAGCAGAGT, rv; TTTGTGTTTGGACTCTCCCC for GAPDH.

### Electrophoretic Mobility Shift Assay (EMSA)

EMSA was performed according to manufacturer’s instructions of the HSE EMSA kit (Panomics AY1020P, USA). In short, equal amounts of nuclear fractions (4 μg) of heat shocked HL-1 cardiomyocytes with or without calpeptin treatment were obtained as described above. Transcription factor-DNA-probe complexes were allowed to form by incubating the nuclear extracts with a DNA-probe. The DNA probe consisted of a biotin labelled HSE probe (Heat shock consensus element CTGGAATTTTCCTAGA) or an unlabeled HSE probe (cold probe). As a positive control, nuclear extract prepared from a HeLa cell line was used (supplied). Samples were subsequently separated on a 6% non-denaturating polyacrylamide gel and transferred to a positively charged Nylon membrane (Amersham, UK). Proteins were cross-linked by UV crosslinker (Stratagene, The Netherlands). After blocking and washing of the membrane in the supplied buffers, the membrane was incubated with a streptavidin-HRP mixture. The HSF1-HSE complexes were detected by applying the provided detection buffer and subsequent detection with a chemiluminscent imaging system (GeneGnome/GeneTools from SynGene, USA). For control and calpeptin treated cells, a competition assay with unlabeled (cold) probe was performed as control for binding specificity. In both cases addition of the cold probe attenuated the intensity of the observed band, thereby indicating specific binding to the HSE-probe.

### Statistical Analysis

Results are expressed as mean±SEM. Biochemical analyses were performed at least in duplicate. Multiple-group comparisons were obtained by ANOVA, with 1-way ANOVA for nonrepeated measurements. Individual group mean differences were evaluated with the Student t test and Bonferroni correction. All P values were 2 sided. Values of P<0.05 were considered statistically significant. SPSS version 20 was used for all statistical evaluations.

## Results

### Active RhoA suppresses HSP expression

**Fig 1:**
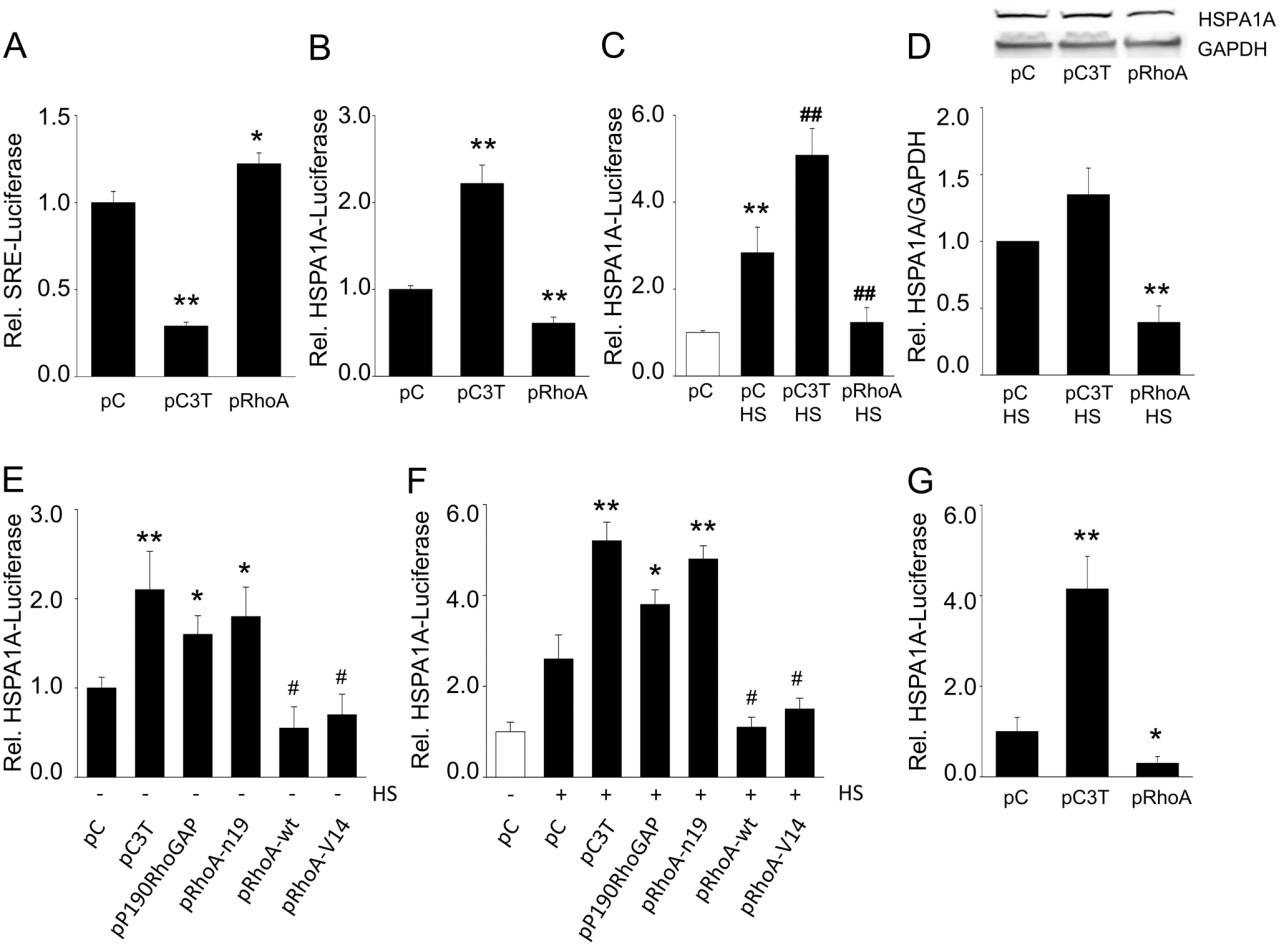
RhoA activation attenuates HSPA1A expression. (A) Relative luciferase expression of a reporter construct driven by the SRE promoter (downstream target of RhoA/ROCK signaling) in HL-1 cardiomyocytes transfected with empty plasmid (pC, pcDNA3.1+), C3T exoenzyme plasmid (pC3T) or RhoA-WT encoding plasmid (pRhoA). (B) Relative luciferase expression of a reporter construct driven by the HSPA1A promoter in cardiomyocytes transfected with pC, pC3T or pRhoA. (C) Relative HSPA1A-luc expression in cardiomyocytes transfected with pC, pC3T or pRhoA and subjected to a HS (45°C, 10 min), white bars represent control non-HS, whereas black bars represent HS cells. (D) Top panel shows a representative Western blot with HSPA1A levels of cardiomyocytes transfected with pC, pC3T or pRhoA and subjected to a HS. Below, quantified data of HSPA1A/GAPDH levels for conditions as indicated. (E) Relative luciferase expression of a reporter construct driven by the HSPA1A promoter in cardiomyocytes transfected with empty plasmid pC, pC3T, pP190RhoGAP, pRhoA-n19, pRhoA or pRhoA-v14 without (E) and with (F) HS. (G) RhoA activation attenuates HSPA1A expression in human HEK-293 cells. Relative luciferase expression of a reporter construct driven by the HSPA1A promoter in HEK293 cells transfected with empty plasmid pC, pC3T, or pRhoA. *P<0.05, **P<0.01, ***P<0.001 compared to control pC and ^#^P<0.05, ^##^P<0.01 compared to pC or pC HS. White bar in panel (F) represents control non-HS cells, whereas black bars represent HS cells.

To determine if RhoA signaling affects the HSR, control and heat shocked (HS) HL-1 cardiomyocytes were transfected with a luciferase reporter construct driven by the HSF1 regulated human HSP70 (HSPA1A) promoter (HSPA1A-luc). RhoA activity was inhibited by transfection of the C3-exoenzyme (pC3T) construct, which inhibits the RhoA pathway, or activated by overexpression of RhoA by transfection of a RhoA wild type construct (pRhoA) (S1A and S1B Fig). The effectiveness of these manipulations was validated using co-transfection with a luciferase construct driven by the RhoA-dependent serum response element (SRE) (Fig 1A). pRhoA expression reduced both basal HSPA1A-luc (Fig 1B) and also strongly suppressed the heat-induced increase in HSPA1A-luc expression (Fig 1C). pRhoA also significantly reduced the level of endogenous HSPA1A protein expression in the total fraction of heat-shocked cells (Fig 1D).

Inversely, inhibition of the RhoA pathway by transient transfection of C3T (pC3T, Fig 1A) almost doubled the HSPA1A-luc activity in unstressed cells (Fig 1B) and enhanced activation of the HSPA1A promoter after heat shock (Fig 1C and 1D). However, the increase in endogenous HSPA1A expression levels did not reach significantly increase (P = 0.07) (Fig 1D). In addition, overexpression of a constitutively active RhoA plasmid, RhoA-v14, also reduced HSPA1A-luc expression, even after a heat shock, while it was enhanced by RhoA inhibition in cells transfected with P190RhoGAP (that reduces RhoA activity) or the dominant negative RhoA-n19 (Fig 1E and 1F). The observed RhoA inhibition of the HSR is not limited to HL-1 cardiomyocytes, as comparable findings were observed in human HEK-293 kidney cells, in which RhoA activation reduced HSPA1A-luc expression, and RhoA inhibition by C3T augmented it (Fig 1G). Together, these findings show that the HSR is modulated by the activity of the RhoA pathway even to such extent that HSR activation by external proteotoxic stress can be abrogated.

**Fig 2:**
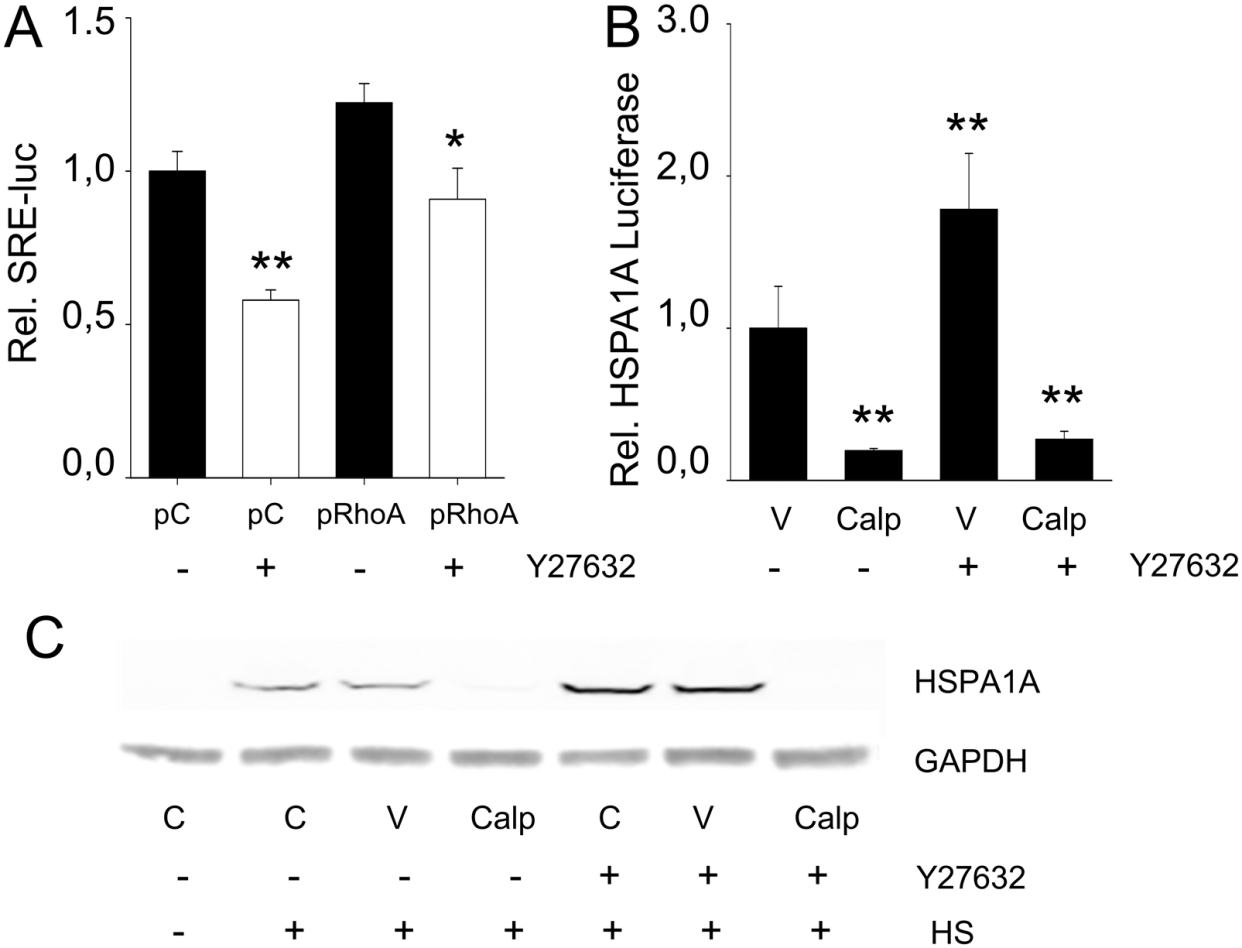
Suppression of the HSR is independent of RhoA’s downstream effector ROCK. (A) Relative SRE-luciferase expression in cells transfected with empty plasmid (pcDNA3.1+, (pC)) or RhoA-WT encoding plasmid (pRhoA) with or without ROCK inhibitor Y27632. (B) Relative HSPA1A-luc expression in cells treated with calpeptin (Calp) compared to control [V] cells with or without ROCK inhibitor Y27632. (C) Representative Western Blot of HSPA1A expression in cells treated with calpeptin (Calp) compared to control [C] cells or DMSO treated cells [V] with or without ROCK inhibitor Y27632. *P<0.05, **P<0.01 compared to control (pC or V).

To explore whether suppression of the HSR by active RhoA was mediated via its common downstream effector ROCK, we tested the effects of its inhibitor Y27632. Adequate inhibition of ROCK by Y27632 was confirmed by the SRE-luciferase reporter (Fig 2A). RhoA was activated by treating the cardiomyocytes with calpeptin (S1B Fig). Nevertheless, Y27632 did not affect suppression of HSPA1A-luc levels by active RhoA (Fig 2B), nor the suppression of HSPA1A expression in normal and heat shocked cells (Fig 2C). Comparable findings were observed for the non-selective ROCK inhibitor H1152P (S2 Fig). This finding indicates that the active RhoA-induced suppression of the HSR is independent of ROCK activity.

**Fig 3:**
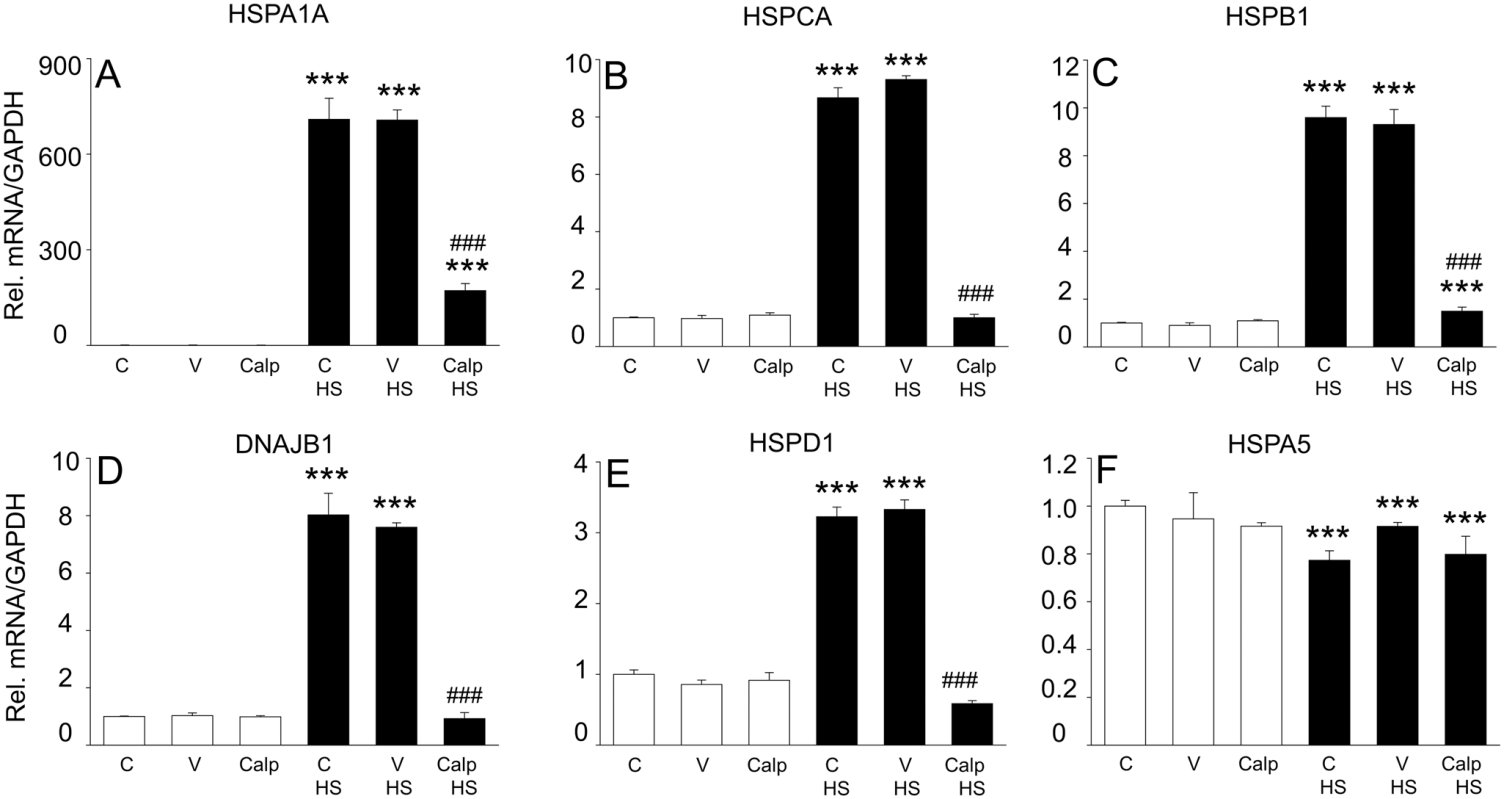
RhoA activation attenuates expression of multiple HSP family members. Cells were non-treated [C], treated with DMSO [V] or calpeptin [Calp] with or without HS (45°C, 10 min) and mRNA levels of (A) HSPA1A, (B) HSPCA, (C) HSPB1, (D) DNAJB1, (E) HSPD1, and (F) HSPA5 were determined by qPCR. White bars represent non-HS cells, whereas black bars represent HS cells. ***P<0.001 compared to control [V] and ^###^ P<0.001 compared to control [V] HS.

To further examine if active RhoA indeed affects the general HSR, mRNA levels of various endogenous HSP members were determined by quantitative PCR, including the HSF1-regulated HSPA1A, HSPCA, HSPB1, DNAJB1, and HSPD1 and the HSF1-independent HSPA5 (Fig 3). In non-heat shocked cells, modulation of RhoA by calpeptin resulted in minor changes in the expression of the HSP mRNAs. In contrast, a mild heat shock significantly induced mRNA expression of all HSF1-dependent HSP genes, which was completely suppressed by active RhoA. As a control we show that active RhoA did not suppress the expression of the ER-resident HSP70 family member HSPA5 (Fig 3F), consistent with HSPA5 being regulated largely independently of HSF1 [22]. These results further emphasize that upon proteotoxic stress, active RhoA suppresses the HSF1 dependent transcription.

Finally, we show that inhibition of the proteasome (by MG-132) [23] or calpain (by PD150606) [24,25] did not suppress the heat-induced activation of the HSR (S3 Fig), meaning that the calpeptin effects were not mediated to either one of these targets.

### RhoA impairs binding of HSF1 to the HSE, which is independent of post-translational modifications

**Fig 4:**
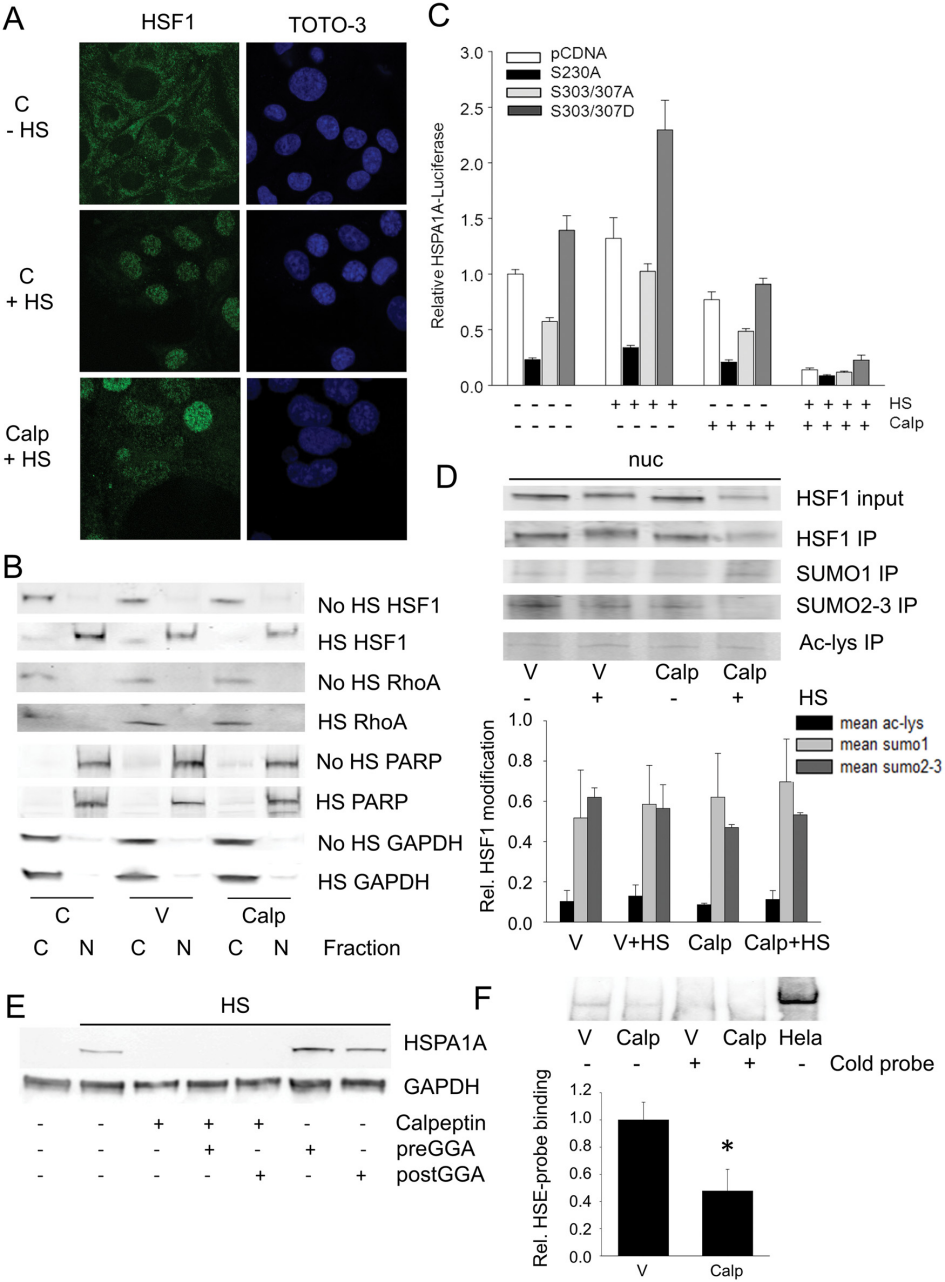
RhoA activation inhibits HSF1 transcriptional activity by suppressing HSF1 binding to HSE, which is independent of HSF1 translocation and post-translational modifications. (A) Nuclear accumulation of HSF1 in response to HS with and without RhoA modulation by calpeptin [Calp] in cardiomyocytes. (B) HSF1 and RhoA levels in TX-100 soluble cytosolic fractions and nuclear-containing fractions of cells with and without HS and RhoA modulation by Calp. The nuclear protein poly-ADP ribose polymerase (PARP) is exclusively present in nuclear containing fractions. C is control non treated cells and V is DMSO (solvent) treated cells. (C) Relative HSPA1A-luc expression in cells transfected with pcDNA3.1+, pS230A, pS303/S307A or pS303/S307D with or without calpeptin (Calp) and subjected to a HS (45°C, 10 min). None of the HSF1 mutants were able to rescue the calpeptin-induced HSPA1A suppression. (D) Top panel: Western blot showing sumoylation (SUMO1 and SUMO2-3) and acetylation status (Ac-lys) of immunoprecipitated (IP) HSF1, obtained from TX-100 nuclear containing fractions of cells treated with or without calpeptin (Calp) and subjected to a HS. Lower panel: Quantified data showing no effect of calpeptin on acetylation and sumoylation levels of HSF1. (E) Representative Western blot of HSPA1A expression in heat shocked cells pre- and post-treated with the HSF1 enhancer GGA with and without calpeptin treatment. GGA results in boosting of the HSPA1A expression after heat shock, but was not able to rescue the calpeptin-induced suppression of the HSPA1A expression. (F) Top panel: EMSA for HSF1 binding to the HSE in response to RhoA modulation and HS, with HeLa nuclear cell extract as a positive control and a competition-assay with a non-labeled HSE probe (cold probe) for DMSO (V) and calpeptin (Calp) treated cells, to determine specific binding to the HSE probe. Lower panel: calpeptin significantly attenuated HSF1-HSE binding compared to V.

As active RhoA suppresses the transcription of all HSF1-regulated HSPs examined, we investigated its action on the main steps of HSF1 transcriptional activation, i.e. nuclear accumulation, post-translational modifications (phosphorylation, acetylation and/or sumoylation), and binding to the HSE. In control cells, HSF1 was mainly located in the cytosol (Fig 4A), but after heat shock, HSF1 accumulates in cell nuclei (Fig 4A) and the TX-100 nuclear-containing fraction (Fig 4B). Activation of RhoA, by calpeptin, did not affect the heat shock-induced HSF1 nuclear translocation (Fig 4A and 4B).

In addition, phosphorylation of HSF1 can modulate its activation [2]. Therefore, the role of RhoA on the phosophorylation status of HSF1 was tested. The heat shock-induced hyperphosphorylation, as evidenced by its decreased mobility on SDS-PAGE [26], was unaffected by RhoA activation (Fig 4B), suggesting that the suppressive effect of active RhoA on HSR is independent on a general change in phosphorylation status of HSF1. To test whether active RhoA influences the specific phosphorylation sites of HSF1, cells were transfected with the HSF1 mutants S303/S307A (which blocks sumoylation of HSF1 at K298), the constitutively phosphorylated S303/S307D (which stimulates sumoylation at K298), or with the control construct, the non phosphorylatable S230A (which blocks HSF1 activation) and subjected to heat shock and calpeptin treatment (Fig 4C, S4A and S4B Fig). As the HSF1 S303/S307 mutants were unable to rescue the active RhoA-induced suppression of the HSR (Fig 4C), sites S303/S307 seem not involved.

Next, we examined if other post-translational modifications of HSF1, i.e. acetylation and/or sumoylation, are modulated by RhoA, such that they can explain the inhibitory effects on HSF1 activation. Hereto, immunoprecipitation (IP) of nuclear HSF1 was performed, since HSF1 was mainly present in the nuclear fractions of cells that underwent heat shock (Fig 4B and 4D). Active RhoA, however, did not affect acetylation, SUMO1, or SUMO2-3 levels of HSF1 in heat shocked cells compared to non-treated heat shocked cells. Our findings thus indicate that the suppression of HSR by active RhoA in cells with proteotoxic stress is independent of a general effect on HSF1 translocation, phosphorylation, acetylation, and sumoylation and the phosphorylation of S303/S307.

Also, we tested whether enhanced activation of HSF1 can rescue RhoA mediated suppression of the HSR, by examining the effects of the HSF1 activator GGA in heat shocked cells in which RhoA was activated by calpeptin treatment. Neither pre- nor post-heat shock treatment with GGA rescued the calpeptin-induced suppression of HSPA1A expression, despite the boosting of HSPA1A expression in control heat shocked cells (Fig 4E). Finally, we asked whether the nuclear accumulated HSF1 actually binds the HSE under conditions of RhoA activation and heat shock. Hereto, we examined the binding of HSF1 from isolated nuclear TX100 fractions to a biotin labelled HSE-probe in heat shocked cells treated with or without calpeptin. In untreated cells, heat shock resulted in a large upward shift of the HSE (S5 Fig, upper band arrowhead), indicative of binding of HSF1 to the HSE-probe. Furthermore, addition of excess unlabeled HSE-probe blocked the mobility-shift of HSE, demonstrating specificity of binding. Importantly, calpeptin treatment significantly reduced the mobility-shift of HSE observed in control heat shocked cells (Fig 4F), suggesting that RhoA inhibits HSF1 effects by reducing its DNA binding. Because RhoA was recently reported to regulate transcription following nuclear translocation [26], we examined whether active RhoA itself might impair HSF1 binding to HSE. However, RhoA was not recovered from the nuclear fractions in control and heat shocked cells treated with calpeptin, despite it being present in the cytosolic fractions of these cells (Fig 4B). Thus RhoA nuclear translocation is absent following RhoA activation and hence cannot explain inhibition of HSF1 binding to DNA.

Taken together, our findings indicate that in cells with proteotoxic stress, active RhoA impairs the ability of HSF1 to bind the HSE in *hsp* genes independent of a (general) effect on its translocation, phosphorylation, acetylation and sumoylation.

### Active RhoA sensitizes cells to proteotoxic stimuli

**Fig 5:**
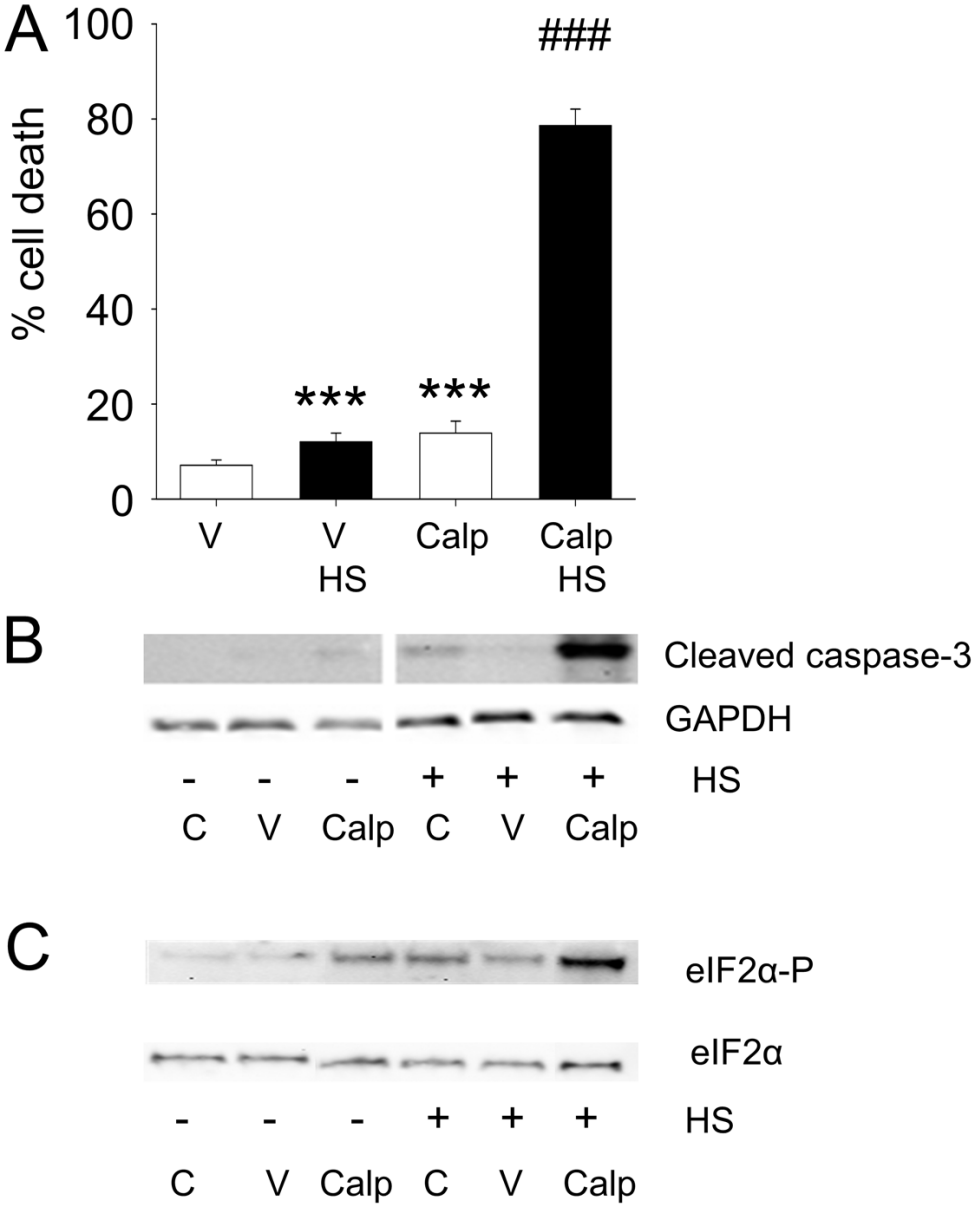
RhoA-induced suppression of the HSR decreases cell stress resistance and induces cell death via apoptosis. (A) Percentage trypan blue positive cells treated with DMSO [V] or calpeptin (Calp) with or without a HS (45°C 10 min) with a 4h recovery period. White bars represent non-HS cells, whereas black bars represent HS cells. (B) Representative Western Blot of cleaved-caspase 3 in control [C], DMSO [V] or calpeptin [Calp] treated cells with or without a HS. (C) Representative Western Blot showing eIF2α 51S phosphorylation and eIF2α levels for conditions as indicated. ***P<0.001 compared to control [V] and ### P<0.001 compared to control [V] HS.

To examine the consequence of active RhoA on cellular sensitivity to stress, cells were subjected to a sublethal HS followed by a trypan blue uptake measurement 4h after HS (Fig 5A). In control cells, activation of RhoA by calpeptin resulted in a small, but significant, increase in cell death (Fig 5A). However, calpeptin treatment grossly enhanced cell death in heat shocked cells (Fig 5A), also illustrated by enhanced caspase-3 cleavage and hyperphosphorylation of eIF2α in heat shocked cells (Fig 5B and 5C), indicative of a stronger heat damage response with a permanent arrest of protein translation [27,28]. These findings show that suppression of the HSR by active RhoA has functional consequences such that it sensitizes cells to proteotoxic stimuli.

## Discussion

The current study identifies active RhoA as a suppressor of the HSR by impairing the binding of HSF1 to the HSE in the promoter region of *hsp* genes. This impairment of binding is not caused by previously recognized mechanisms, including the loss of translocation of the HSF1 from the cytosol to the nucleus, nor by general changes in phosphorylation, acetylation, and sumoylation levels of HSF1 and the phosphorylation of HSF1 at S303/S307. In addition, suppression of the HSR by active RhoA is independent of its canonical signaling via ROCK and its direct interaction with the transcription machinery, since RhoA did not translocate to the nucleus. Our disclosure of active RhoA as a suppressor of the HSR, both representing major stress activated pathways, may profoundly change our understanding of the orchestration of the stress response under normal and pathological conditions.

RhoA mediated suppression of the HSR sensitizes cells for proteotoxic stress and might therefore be of relevance in various age-related diseases, including cardiac and neurodegenerative diseases. In age-related diseases, protein misfolding and aggregation are caused by cellular proteins that are challenged throughout life by a multitude of factors. It has been shown that HSPs play a role in preventing and elimination of proteotoxic aggregates [29,30], but that in ageing cells the HSR system seems to fail as evidenced by accumulation of protein aggregates [29]. Thus, RhoA activation may represent one of the mechanisms leading to the failure of ageing cells to mount an adequate HSR. Such a mechanism may also apply in other conditions, e.g. in case of the tachycardia atrial fibrillation. In atrial fibrillation, disease progression coincides with both the induction of RhoA activity and the lack of cardiac cells to mount an adequate HSR to address the atrial fibrillation-induced damage [8,11]. Consequently, our current observation suggests that inhibitors of RhoA have beneficial effects in atrial fibrillation by preserving the HSR. In particular, restoration of the HSR may be of clinical interest to increase the success of pharmacological and electrical cardioversion to a normal sinus rhythm. In addition to atrial fibrillation, the HSR suppressive effects of RhoA are thought to contribute to disease progression in age-related neurodegenerative diseases such as Alzheimer’s and Parkinson’s disease. Interestingly, also in these neurodegenerative diseases activation of RhoA has been implied [31], suggesting that the RhoA-induced suppression of the HSR may represent a more general feature of diseases related to proteotoxicity.

In addition to a role of active RhoA in disease progression, RhoA may also represent a key pathway in the propagation of HSR activation between cells or tissues. This so-called cell non-autonomous induction of HSR, i.e. a form of intercellular communication by which stress sensed in one tissue is communicated to another tissue and induces a HSR, was initially reported in *C*. *elegans* [32,33]. The cell non-autonomous induction of the HSR involves both neurons and systemic factors released by neurons, as recently has been suggested for serotonin [34]. Interestingly, serotonin and other systemic factors such as endothelin-1, angiotensin II, noradrenaline, and acetylcholine, modulate the activity of RhoA either by direct activation of RhoA via the RhoGEF AKAP-Lbc [35], by redirecting prototypical Gi-coupled receptors from Rac1 to RhoA activation [36] or by sequestration of p190RhoGAP [37]. It is conceivable that, upon activation of the HSR, the release of systemic factors modulate the activation of RhoA in distant cells, thus influencing their HSR.

In summary, the current study identifies a previously undisclosed action of active RhoA consisting of an HSF1 dependent inhibition of the HSR. While active RhoA did not preclude the nuclear accumulation of HSF1 upon proteotoxic stress, it impaired HSF1 binding to the HSE in the promoter sequence of *hsp* genes, resulting in the suppression of HSP expression and subsequent sensitization to cell death. These findings identify a novel role for active RhoA as suppressor of the HSR in stressed cells and disclose its prominent role in the decision between cell survival and cell death. However, further research is necessary to elucidate whether RhoA activation, by systemic factors, also attenuates the HSR and whether this mechanism is also applicable in other cells/organs.

## Supporting Information

S1 Fig RhoA protein and activity levels after transient transfections and chemical activation of RhoA. (DOCX)

S2 Fig Suppression of the HSR is independent of RhoA’s downstream effector ROCK. (DOCX)

S3 Fig Effective suppression of the HSR by calpeptin and not MG132 or PD150606 treatment. (DOCX)

S4 Fig HSF1 expression levels and transient transfection efficiency in HL-1 cardiomyocytes. (DOCX)

S5 Fig EMSA for HSF1 binding to the HSE. (DOCX)
