# Supplementary material for: RhoA Activation Sensitizes Cells to Proteotoxic Stimuli by Abrogating the HSF1-Dependent Heat Shock Response
Source: PLoS One. 2015 Jul 20;10(7):e0133553. doi: 10.1371/journal.pone.0133553 (PMC4508109; doi:10.1371/journal.pone.0133553)
Supplement: S1 Fig — (DOCX) [file pone.0133553.s001.docx]

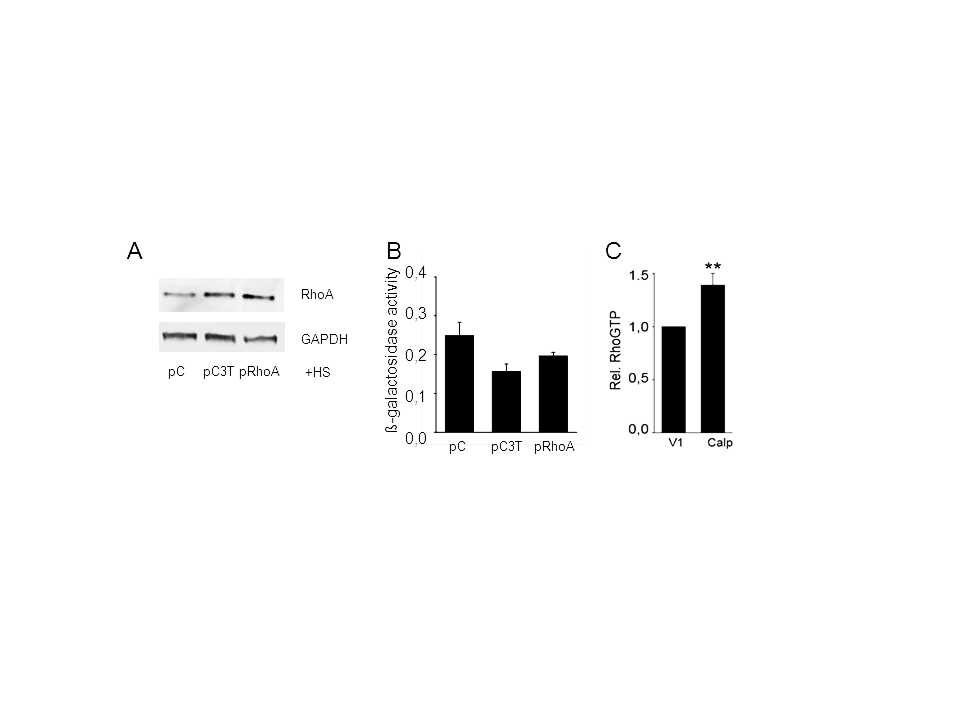


**S1 Fig. RhoA protein and activity levels after transient transfections and chemical activation of RhoA.** A) RhoA protein levels in cells transfected with empty plasmid (pcDNA3.1+, (pC)), C3T exoenzyme plasmid (pC3T), or RhoA-WT encoding plasmid (pRhoA). B) Transient transfection efficiency was determined by co-transfection of cells with pC, pC3T or pRhoA-WT with the β-galactosidase construct (PDM2-LacZ). No differences in transfection efficiency were observed between the groups. C) Relative Rho-GTP levels, as determined by Rho GLISA, for cells treated with calpeptin (Calp, 1 U/ml for 20 min) compared to cells treated with vehicle (DMSO).
