# Supplementary material for: RhoA Activation Sensitizes Cells to Proteotoxic Stimuli by Abrogating the HSF1-Dependent Heat Shock Response
Source: PLoS One. 2015 Jul 20;10(7):e0133553. doi: 10.1371/journal.pone.0133553 (PMC4508109; doi:10.1371/journal.pone.0133553)
Supplement: S2 Fig — (DOCX) [file pone.0133553.s002.docx]

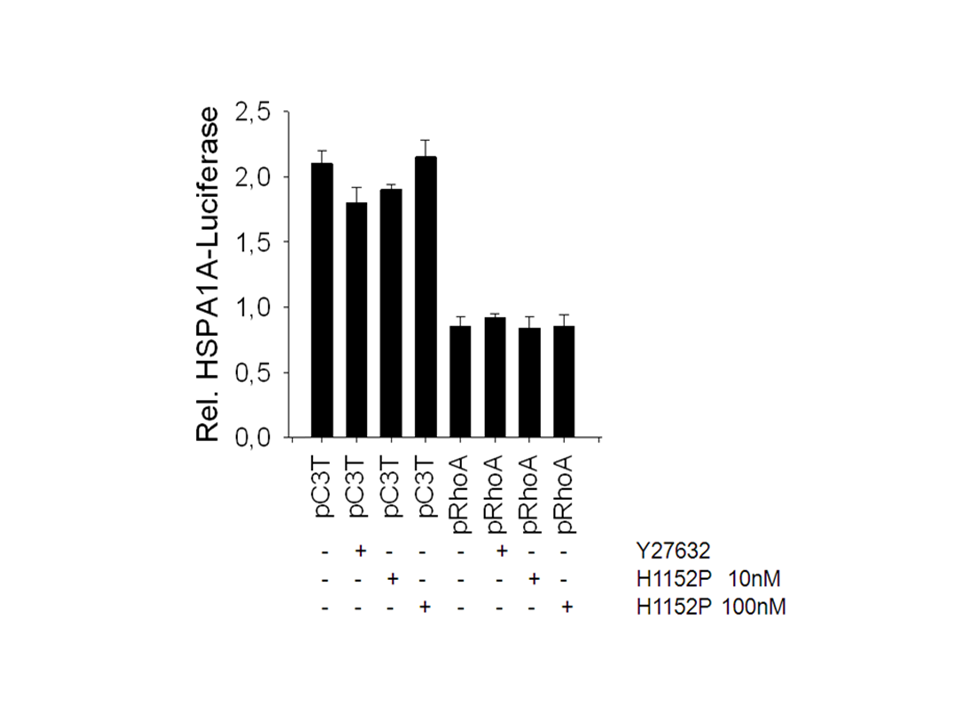


**S2 Fig. Suppression of the HSR is independent of RhoA’s downstream effector ROCK.** Relative HSPA1A-luc expression in cells transfected with pC3T or RhoA-WT encoding plasmid (pRhoA) with or without ROCK inhibitor Y27632 or H1152P at concentrations as indicated. None of the ROCK inhibitors revealed an effect on the RhoA induced suppression of the HSPA1A expression.
