# Supplementary material for: RhoA Activation Sensitizes Cells to Proteotoxic Stimuli by Abrogating the HSF1-Dependent Heat Shock Response
Source: PLoS One. 2015 Jul 20;10(7):e0133553. doi: 10.1371/journal.pone.0133553 (PMC4508109; doi:10.1371/journal.pone.0133553)
Supplement: S3 Fig — (DOCX) [file pone.0133553.s003.docx]

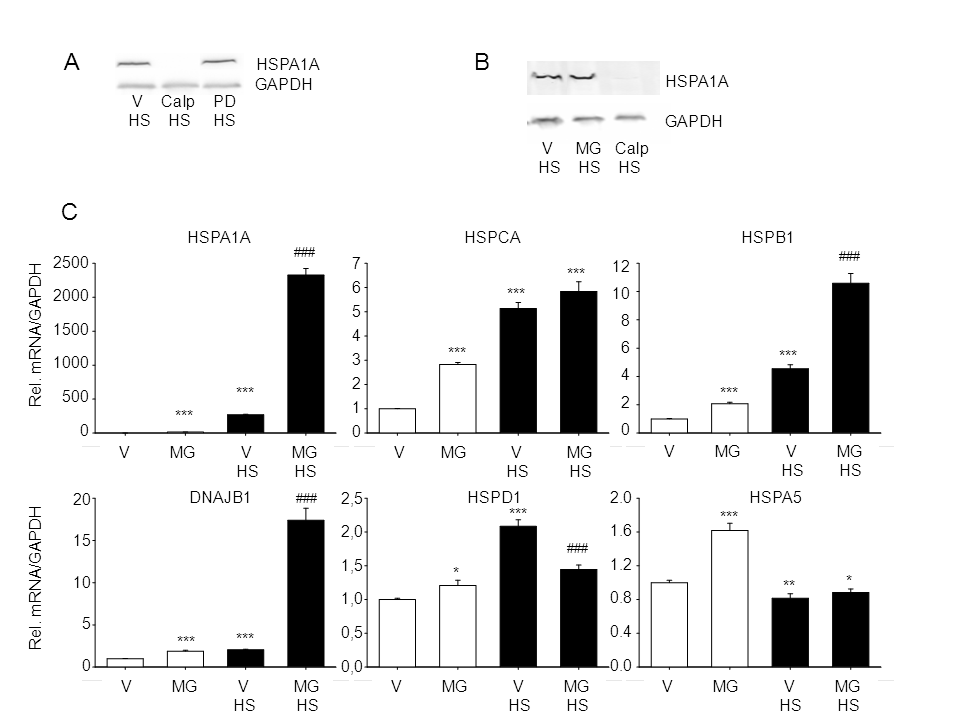


**S3 Fig. Effective suppression of the HSR by calpeptin and not MG132 or PD150606 treatment**. A) Representative Western blot of HSPA1A and GAPDH for cells treated with DMSO (V), calpeptin (Calp) or calpain inhibitor PD150606 (PD) after HS (10 min 45ºC). B) Representative Western Blot of HSPA1A and GAPDH for cells treated with DMSO (V), Calp or proteasome inhibitor MG132 (MG) after HS. C) Quantified qPCR data of HSPA1A, HSPCA, HSPB1, DNAJB1, HSPD1 and HSPA5 mRNA levels for cells treated with DMSO (V) or MG132 (MG) with or without a HS. White bars represent control non-HS cells, whereas black bars represent HS cells. *P<0.05, **P<0.01, ***P<0.001 compared to control (V) and ### P<0.001 compared to control (V) HS.
