# Supplementary material for: RhoA Activation Sensitizes Cells to Proteotoxic Stimuli by Abrogating the HSF1-Dependent Heat Shock Response
Source: PLoS One. 2015 Jul 20;10(7):e0133553. doi: 10.1371/journal.pone.0133553 (PMC4508109; doi:10.1371/journal.pone.0133553)
Supplement: S4 Fig — (DOCX) [file pone.0133553.s004.docx]

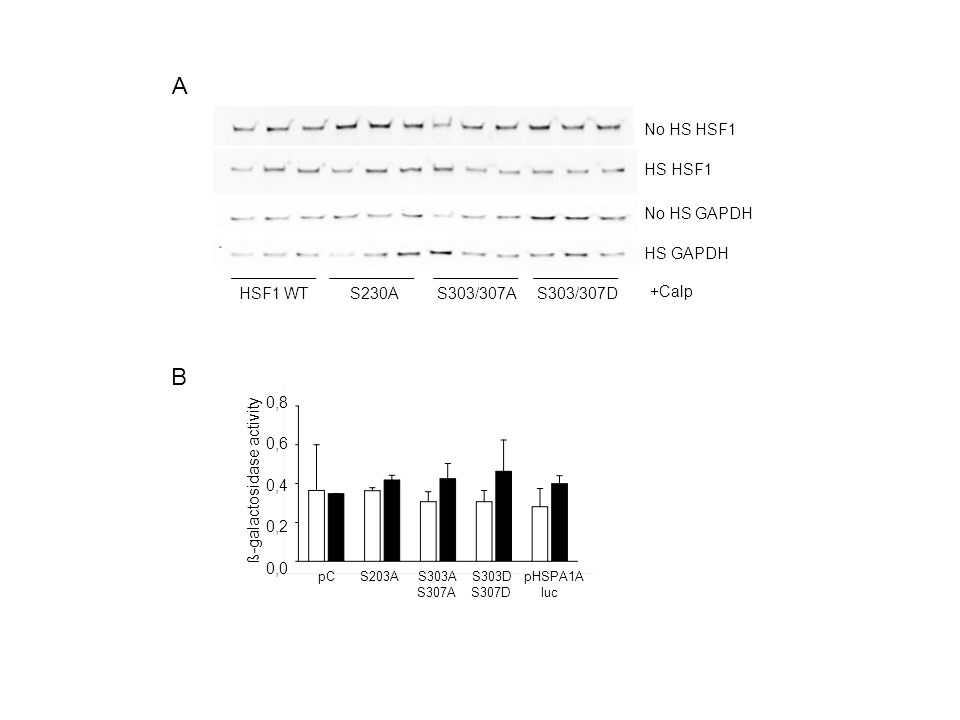


**S4 Fig. HSF1 expression levels and transient transfection efficiency in HL-1 cardiomyocytes**. A) Western blot showing HSF1 expression levels for the conditions as indicated. B) Transient transfection efficiency was determined by co-transfection of cells with pcDNA, S203A, S303/307A, S303/307D, or pHSPA1A-luc, together with the β-galactosidase construct (PDM2-LacZ). No differences in transfection efficiency were observed between the groups.
