# Supplementary material for: RhoA Activation Sensitizes Cells to Proteotoxic Stimuli by Abrogating the HSF1-Dependent Heat Shock Response
Source: PLoS One. 2015 Jul 20;10(7):e0133553. doi: 10.1371/journal.pone.0133553 (PMC4508109; doi:10.1371/journal.pone.0133553)
Supplement: S5 Fig — (DOCX) [file pone.0133553.s005.docx]

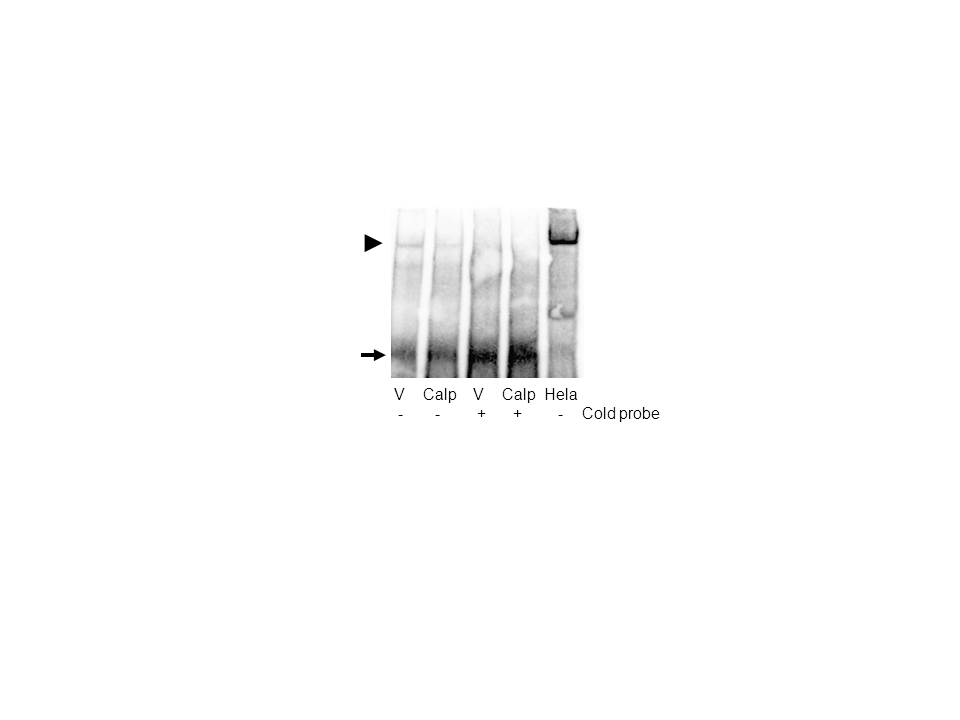


**S5 Fig. EMSA for HSF1 binding to the HSE.** EMSA for HSF1 binding to the HSE in response to RhoA modulation and HS, with HeLa nuclear cell extract as a positive control and a competition-assay with a non-labeled HSE probe (cold probe) for DMSO (V) and calpeptin (Calp) treated cells, to determine specific binding to the HSE probe.
